# Supplementary material for: Acceptance and compliance with micronutrient powder (MNP) among children aged 6–23 months in northern Nigeria
Source: PLOS Glob Public Health. 2022 Oct 17;2(10):e0000961. doi: 10.1371/journal.pgph.0000961 (PMC10022258; doi:10.1371/journal.pgph.0000961)
Supplement: S1 Dataset — (ZIP) [file pgph.0000961.s008.zip › S8_Dataset/Direct Observations.zip!/Direct Observations/010715_K_BK_AD_2_DO.pdf]

1.1.1 Instrument F –Direct Observation Form

<<Instrument F Direct Observation Form>>
PI Name: PragyaMathema,Stephen Kodish
Study Title: Formative research to develop an infant and young child feeding (IYCF) intervention with micronutrient powder (MNP) for children aged 6 – 23 months
PI Version/Date: Version1.0/January 24, 2015

Date: 01/07/15 Location: Gorun Chindo

Data Collector: Amina Duniya

Main goals of this direct observation are to answer the following questions:

1. In this household, what would be the best way(s) to give nutritional supplements to young children? Why?

2. In this household, describe the food sharing that you observe throughout the meal. Are there any predictable patterns?

3. In this household, who is favored and how is this favoritism expressed during meal preparation and eating?

Household Composition Table (Fill out as you do data collection)

INITIALS	SEX (M/F)	AGE (estimated)	Relationship to household	Description
BM	F	19yrs	Mother	Dark wearing a blue and orange wrapper and a blue head tie
BB	M	11months	Baby boy	No hair on his head wearing a yellow top and shorts
GM	F	40yrs	Grand mother	Slim and dark in complexion wearing blue and white wrapper
OW	F	25yrs	Second wife	A bit plumby and dark in complexion wearing blue and yellow top with a blue and white wrapper
BG	F	2yrs	Daughter	Dark all back hair style on her head
SD	F	8yrs	Step daughter to mother	Slim light and tying a multi colour wrapper on her chest
SS	M	4yrs	Step son to BB's mother	Dark wearing dirty black trouser no shirt

TIME	ACTOR INITIALS	RECIPIENT INITIALS	WHAT HAPPENED	FOOD	Reactivity
10:00	BM	BB	BM pounding and carrying BB on her back. She is breast feeding the baby.	maize	
10:05	GM		Still pounding		
10:10	BB	BM	Crying for BM to carry him, carries BB from the ground and sits him on a stool next to her		
10:15	BM	GM	Pounding maize and discussing together while BB is crying		
10:20	BM	BB	Picks him up and keeps him on her hips		
10:25	BM		Still pounding maize		
10:30	GM		Leaves BM to pound alone		
10:35	BM	BG	Ask BG to fetch some water for her		
10:40	BB		Starts crying		
10:45	BM	BB	Sits close to me and starts breastfeeding the BB	Breast milk	They both turn and look at me talking
10:50	BM		Goes to GM and they pounding		
10:55	SD		Comes and carries BB		

TIME	ACTOR INITIALS	RECIPIENT INITIALS	WHAT HAPPENED	FOOD	Reactivity
11:00	BG	BM	Comes to BM and starts crying too		
11:05	BB	BM	Playing on the mat with BM		
11:10	BG	BM	Crying and trying to get on BM's laps		
11:15	BM	BB	Breast feeding the baby	Breast milk	
11:20	SS		Comes to sit on the mat with BB, SD and GB, also looking at me		Smiling at me
11:25	SD	BB	Uses her teeth to cut the water melon for BB and gives him water melon	Water melon	
11:30	BM		Gets up from mat and goes to GM		
11:35	BB		Still playing on the mat with SD and BB		
11:40	BB		Playing with SD		
11:50	BG		Gets up and goes to GM room. BB still playing		
11:55	BB		Still playing and urinating on himself while BM is sleeping		
12:00	BB	BM	BB touching BM's head trying to wake her up		
12:05	BM	BB	Wakes up and starts playing with BB		
12:10	BM	BB	Still playing with BB		
12:15	BB		Starts crying		
12:20	BM	BB	He is down with BB and starts breast feeding	Breast milk	
12:30	BM	BB	Removes the nipples from BB's mouth who is already fast asleep		
12:35	BB,BM		sleeping		
12:40	BM		Wakes up and goes to GM's room		
12:45	BB		Still sleeping		

TIME	ACTOR INITIALS	RECIPIENT INITIALS	WHAT HAPPENED	FOOD	Reactivity
12:50	BM		Comes out of GM's room and perform ablution for prayers		
12:55	BM		Goes back to the mortal and continue pounding maize	maize	
1:00	BM		Still pounding while BB is sleeping		
1:05			Finishing pounding maize and comes to lie down with BB		
1:10	BB		Wakes up from sleep and begin to look for BM		
1:15	BM		Comes back and sits on the mat playing with BB		
1:20	BB		Playing on his own		
1:25	BM	BB	Tries to feed BB with kunu gero but he refuses	Kunu gero	
			BM tries again to feed him and he finally accepts	Kunu gero	
1:35	BM	BB	BB is poo poo on the floor and BM washes his bum bum		
1:40	BM,BB		Throw the poo poo over the fence, BB begins to cry and the BM too BB and breast feed him	Breast milk	
1:45	BM	BB	Still breast feeding	Breast milk	
1:50	BG	BM	Gave BM a cup asking her to give her kunu to drink		
1:55	BB,BM		Gets up and goes to her room and BB craws after her		
2:00	OW	BB	Gave BB rice mixed with moringa leaves to eat	Rice with moringa leaves	
2:05	BB		Feeds himself with the food BM gives him, also BG gets hers and sat to eat, BB finishes her food and begins to eat BB's own		
2:10	BM	BG	Stops her from eating BB's food and BG starts to play with her plate		
2:15	BM	BB	BM is trying to get him to eat more	Rice	

TIME	ACTOR INITIALS	RECIPIENT INITIALS	WHAT HAPPENED	FOOD	Reactivity
			rice but he refuses	and moringa leaves	
2:20	BM		Carries BB plate and sweep the food that fell on the mat		
2:25	BB	BM	Continues to play on BMs body		
2:30	BM	BB	Breast feeding the BB		
2:35	BB		Finishes, and crawl away to play		
2:40	BM		Goes to fetch water from the well		
2:45	BG	BB	Comes into the compound and begins to play with the BB		
2:50	BB	BM	Crawl to the bathroom to meet BM		
2:55	BM		Perform ablution in other to pray		
3:00	BM		Still praying		
3:05	BB		Goes to BM who has just finished praying and continues to pray. BM removes BB clothes completely allows him to continue playing		
3:10	BB	BM	Crawl towards BM and starts to cry		
3:15	BM	BB	Bathe BB with soap, sponge and water		
3:20	BM	BB	Dresses BB and breast feed him	Breast milk	
3:25			BM gives moringa leaves (boiled) in a plate to eat	Moringa leaves	
3:30			Eat his food by himself under BM watchful eyes		
3:35	BB		Playing on a mat		
3:40	BM	BB	Puts BB on her back and goes to GM's room		
3:45	BM		Fetches pounded maize and washes it.	Maize	

TIME	ACTOR INITIALS	RECIPIENT INITIALS	WHAT HAPPENED	FOOD	Reactivity
3:50	BM	SD	Put all of the maize in a basket and gives SD for grinding		
3:55			Sitting on the mat with BB still on her back		
4:00	BB		Struggles to come down from BM back to starts playing on the mat		
4:05	SD		Goes to the well to fetch water with BB on her back		
4:10	SD		Still carrying BB		
4:15	SD	BB	Pours some of the milk in BB's mouth	Milk	
4:20	BB		BB playing		
4:25	BB	BM	Crawl on BM's laps and begins to suck breast	Brest milk	
4:30	BB		Falls to the ground and begin to cry		
4:35	BB		Finishing sucking the breast and comes down to play		
4:40			Still playing on the mat		
4:45	BM	BB	Drops him on another mat to play		
4:50	BM		Goes to the well to fetch water		
4:55			Tries to make fire for cooking		
5:00			Resumes trying to make fire		
5:10	BM	BB	Goes into the bedroom with BB on her back		
5:15	BM		Washes a cup and fetch kunu gero for me to drink	Kunu gero	
5:20			Separates the flour from the pellet		
5:25			Finished sieving and put the pellet back into the mortal an begin to pound again		
5:30	BM	BG	BG comes into the compound and told BM to her buttocks		

TIME	ACTOR INITIALS	RECIPIENT INITIALS	WHAT HAPPENED	FOOD	Reactivity
5:35	BM		Brought the pellet back to her		
5:40			Carries empty pot to wash		
5:45	BM		Gets more dry sticks to make fire		
5:50			Adds hot charcoal to dry sticks and keeps blowing with a flat plate		
5:55	BM	BB	Drops BB on the mat to sleep and continues to set fire		
6:00	BB	BM	Crawls to BM and she picks him up		
6:05	BM	BB	Begins to cry and BM puts him to breast her	Breast milk	
6:10	BM		Gets more dry sticks and add to the fire		
6:15			Checks the water in the fire		
6:20			Sieves the maize pellet again		
6:25	BM		Looks for more dry sticks again to add to the fire		
6:30			Washes the bowl used in mixing the paste		
	BB		Still playing alone close to BM		

Primary Questions (Complete <u>after</u> you complete data collection):

From what you have seen observing THIS HOUSEHOLD, please answer the following questions in detail:

Question	Write your <u>detailed</u> response here
1. In this household, what would be the best way(s) to	Through the mother because the mother is the person that supervises the child's during meals

give nutritional supplements such as an MNP to young children to ensure compliance?<br><br>Why?	
2. In this household, who is favored during meal preparation or feeding?<br><br>How is this favoritism expressed?	The BB is favoured the most because the bigger sister is given her plate and told to eat on her own without supervision while the younger one who has his own plate too is feed by the mother.<br>The quantity of food between the two children is almost the same too
3. How could we promote the MNP supplement for this family that would attract their attention?<br><br>Who would they listen to?	The MNP supplement can be promoted through the household head because he is the person who has the final decision in the house. The village head is also very important because the husband must listens to him as his leader
4. In this household, what would be the best ways to promote the MNP but limit how much it would be shared?<br><br>Why?	The best way is to instruct the mother properly on how to keep the properly on how to keep the product away from other children.<br>I noticed that she kept the product inside her bedroom where it is only her husband and daughter that are allowed to go in and when I asked her whether she shared the MNP with others she said no, and I said why? She said because the MNP sachet is meant for the boy only
5. What other issues and concerns did you see with this family that are important for us to consider with regard to introduction of an MNP?<br><br>Why?	The hygienic condition of the plates/cups used in feeding the baby is bad because I noticed that the mother does not wash the plates before putting food in it and<br>Secondly, she does not wash her hands before feeding the child under observation
6. Based on your experience observing this child today, do you believe reactivity negatively impacted your ability to see the food-related behaviors of the child?	I do not think reactivity affected my ability to see food related behaviours because I noticed that the 3yrs old daughter wherever she wanted food or kunu from the mother, she first of all brings her plate/cup before requesting for it. This shows that she is used in eating alone with her own plate/cup

Please explain your answer in detail.	

NB: Before my arrival the MNP had already been given to the BB
